# Safety and Efficacy of Biologic Therapies (Ustekinumab and Vedolizumab) in the Treatment of Inflammatory Bowel Disease (IBD): A Systematic Review

**DOI:** 10.7759/cureus.48338

**Published:** 2023-11-06

**Authors:** Hafsa Ashraf, Adiprasad Bodapati, Ayesha Hanif, Donatus K Okafor, Gitika Katyal, Gursharan Kaur, Safeera Khan

**Affiliations:** 1 Internal Medicine, California Institute of Behavioral Neurosciences & Psychology, Fairfield, USA; 2 Medicne, California Institute of Behavioral Neurosciences & Psychology, Fairfield, USA; 3 Research, California Institute of Behavioral Neurosciences & Psychology, Fairfield, USA

**Keywords:** ibd, remission, treatment, ustekinumab, vedolizumab

## Abstract

Inflammatory bowel disease (IBD) is a group of chronic disorders, including Crohn's disease (CD) and ulcerative colitis (UC), that contribute to inflammation of the gastrointestinal tract, manifesting as bloody diarrhea, fecal urgency, bloating, cramping, and weight loss. IBD manifests as an exacerbation of these symptoms, which medications with high side effect profiles can manage; consequently, many novel therapies, including biologics such as ustekinumab and vedolizumab, have been developed over the years. This systematic review aims to assess the safety and efficacy of ustekinumab and vedolizumab in treating inflammatory bowel disease based on a comprehensive analysis of relevant studies. A thorough literature search was conducted to identify randomized controlled trials, post hoc analyses, case reports, observational cohorts, and meta-analyses involving ustekinumab and vedolizumab as treatment in IBD patients. The selected studies were critically evaluated for their methodology, patient characteristics, and outcomes. The analysis involved twelve distinct studies investigating the impact of ustekinumab and vedolizumab on individuals afflicted with inflammatory bowel disease (IBD). The findings revealed a notable trend: ustekinumab displayed a propensity for yielding higher rates of clinical remission in patients with ulcerative colitis (UC). Moreover, one study underscored substantial reductions in endoscopic disease activity in patients with Crohn's disease (CD) who were on ustekinumab. Similarly, ustekinumab exhibited promising outcomes in CD patients, including swift ultrasound responses and the achievement of transmural remission, particularly among those who were new to biologic treatments. In line with this, vedolizumab demonstrated early and considerable symptomatic improvements when used to treat both UC and CD patients. While both biologics showed promising results in inducing and maintaining remission, cautious monitoring is warranted due to the potential adverse events observed in some cases. Further research with larger sample sizes and longer follow-up periods is needed to establish a comprehensive understanding of the medications' effects on IBD patients.

## Introduction and background

Inflammatory bowel disease (IBD) comprises a group of chronic, immune-mediated conditions with multifactorial etiology, the two major phenotypes being Crohn's disease (CD) and ulcerative colitis [1]. It is estimated that the prevalence of inflammatory bowel disease is approximately 1 million among residents in the United States and 2.5 million among residents in Europe, and an apparent increase in its prevalence has been observed in recently industrialized nations in Asia, South America, and the Middle East, and has subsequently developed into a worldwide ailment with increasing frequency across all continents [2]. Since there is no definite cure for IBD, many conventional nonbiologic therapies like aminosalicylates, corticosteroids, and immunomodulators like methotrexate have been used to control symptoms. But, over the last two decades, significant advancements have been made in managing IBD, including the introduction of novel biologic therapies [3]. Among these, vedolizumab [3] and ustekinumab have emerged as promising treatment options, demonstrating efficacy in inducing and maintaining remission in patients with IBD.

Ustekinumab is a human monoclonal antibody directed against the p40 subunit of IL-23, approved for patients with IBD. At the same time, vedolizumab (VDZ) is a humanized, gut-selective monoclonal IgG1 antibody that targets the heterodimer α4β7 integrin, inhibiting migration and leukocyte adhesion [4]. Both drugs belong to a group of drugs called biologic therapies [5]. Vedolizumab and ustekinumab, the newer biologic therapies, have a better safety profile than immunomodulators [6]. The safety and efficacy of ustekinumab and vedolizumab in treating IBD have been investigated in numerous clinical trials and real-world studies.

However, to date, a comprehensive synthesis and analysis of the available evidence on the use of these biologic agents in IBD treatment are lacking. Therefore, this systematic review aims to address this gap by critically evaluating and synthesizing the existing literature on the safety and efficacy of ustekinumab and vedolizumab in managing IBD.

We aim to provide a robust and comprehensive overview of the current evidence base by employing a systematic approach. This systematic review will encompass a comprehensive examination, meticulous selection, and critical evaluation of pertinent research studies in accordance with established protocols such as the Preferred Reporting Items for Systematic Reviews and Meta-Analyses (PRISMA) statement. The selected studies will encompass randomized controlled trials, observational studies, systematic reviews/meta-analyses, and case reports, ensuring diverse evidence sources. 

## Review

Methods

The systematic review was carried out in accordance with the Preferred Reporting Items for Systematic Review and Meta-Analysis (PRISMA) 2020 [7].

Search strategy

We used PubMed, PubMed Central, Medline, Science Direct, and Multidisciplinary Digital Publishing Institute (MDPI) to search for the relevant literature on the topic. We used the keywords, i.e., IBD, treatment, vedolizumab, ustekinumab, safety, efficacy in combination, and Booleans AND, OR, to search all databases. The keywords and the following strategy were developed to search for relevant literature in PubMed's Medical Subject Headings (MeSH) database: (("inflammatory bowel diseases/drug therapy"[Majr] OR "inflammatory bowel diseases/prevention and control"[Majr] AND "vedolizumab" [Supplementary Concept] AND "ustekinumab/adverse effects"[MeSH] OR "ustekinumab/therapeutic use"[MeSH].

Table 1 presents an overview of the databases employed in this study, together with the corresponding quantities of papers identified for each respective database.

**Table 1:** Keywords/strategy used and the number of identified papers IBD - inflammatory bowel disease; MeSH - Medical Subject Headings; MDPI - Multidisciplinary Digital Publishing Institute; PMC - PubMed Central

Keywords/ search strategy	Database used	Number of papers
IBD treatment AND vedolizumab AND remission AND ustekinumab	Science Direct	82
("inflammatory bowel diseases/drug therapy"[Majr] OR "inflammatory bowel diseases/prevention and control"[Majr] AND "vedolizumab" [Supplementary Concept] AND "ustekinumab/adverse effects"[MeSH] OR "ustekinumab/therapeutic use"[MeSH])	PubMed MeSH database	359
IBD treatment AND vedolizumab AND ustekinumab	PubMed	24
IBD treatment AND vedolizumab AND ustekinumab	MDPI	10
((IBD [Text Word]) AND (vedolizumab [Text Word])) AND (ustekinumab [Text Word])	PMC	22

Inclusion and Exclusion Criteria

We selected the articles and literature published in the last five years; we included papers written only in the English language. Our selection criteria were limited to articles and research exclusively involving adult human subjects. 

Articles for which the complete text of the papers was not extracted were eliminated from the analysis. Additionally, the review did not include research that focused on pediatric age groups. Grey literature and proposal articles were also not included.

Selection Process

We transferred the selected articles to the endnote and removed duplicate papers. Each paper was individually screened through titles and abstracts by HA (first author). In the event of a dispute regarding eligibility, the concerns were deliberated with all remaining co-authors and ultimately resolved by mutual agreement. The papers included in the shortlist underwent a subsequent evaluation process, wherein the whole text of each item was carefully analyzed. Only those articles that were deemed relevant were further considered for assessment. Applying inclusion and exclusion criteria resulted in selecting articles that met the specified criteria and narrowing down the list.

Quality Assessment of Studies

The articles selected for further consideration underwent an evaluation of their quality using appropriate instruments for quality appraisal. The screening process required the participation of all co-authors. The quality of observational studies was evaluated using the Newcastle-Ottawa method, whereas the Assessment of Multiple Systematic Review (AMSTAR) tool was employed to examine systematic reviews. The Cochrane Risk of Bias Assessment Tool (version 2) was employed to evaluate the methodological quality of the randomized controlled trials. The systematic review only included studies that met the criteria for quality appraisal.

Results

Study Identification and Selection

A total of 499 relevant articles were collected from all databases. A total of 30 duplicate articles were excluded before conducting a thorough screening process. These articles were screened by going through titles and abstracts, and then retrieving full-text articles; 29 articles were shortlisted after this process. These 29 articles were put through quality assessment, and 12 articles were then finalized for review. The selection process is shown in the following flowchart.

**Figure 1:**
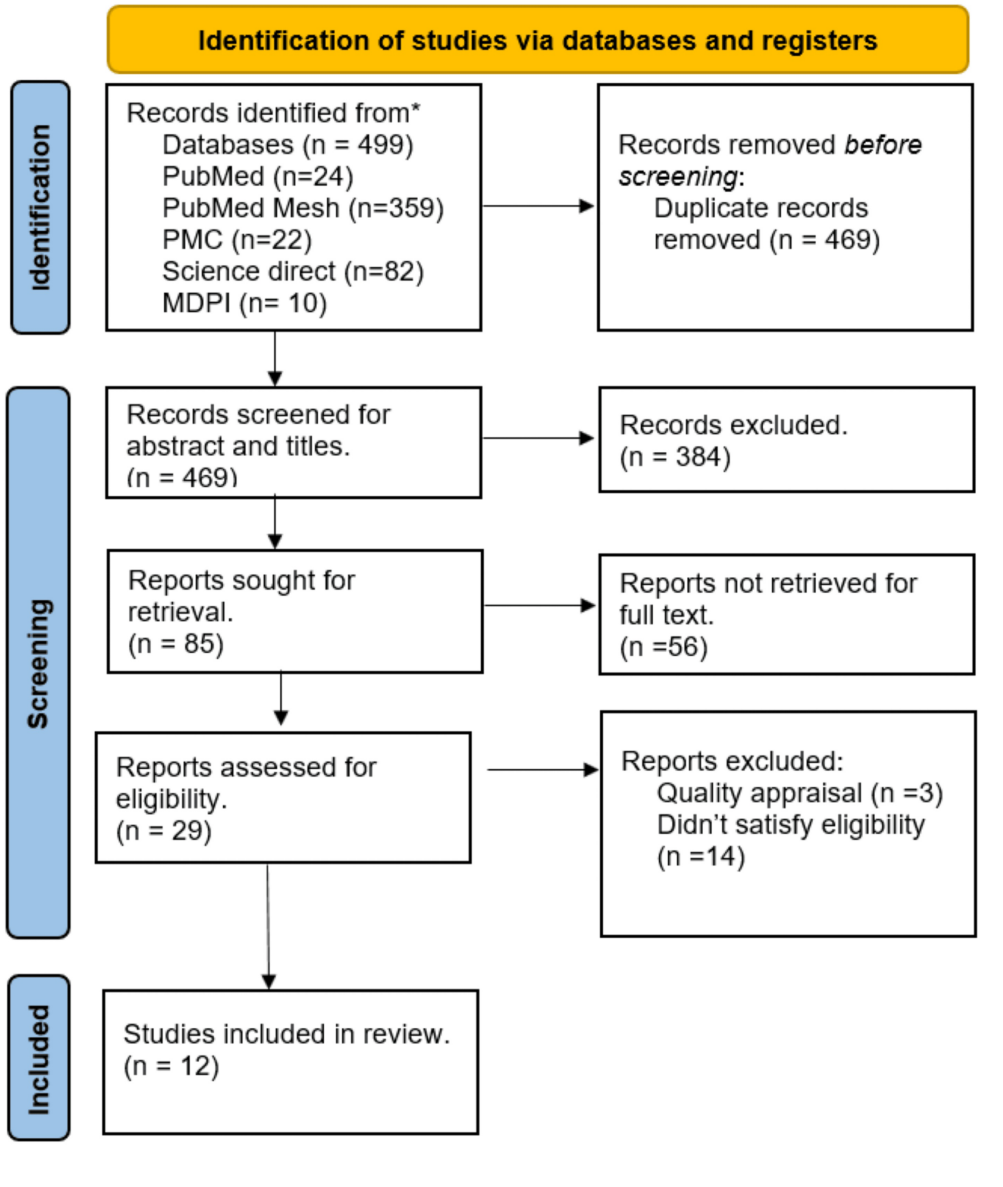
PRISMA flowchart showing the process of article selection PRISMA - Preferred Reporting Items for Systemic Review and Meta-Analysis

The process of selecting the research papers is depicted in Figure 1 of the PRISMA flowchart.

The articles were assessed for eligibility using the relevant quality assessment appraisal tools. A comprehensive analysis was conducted on a set of five randomized controlled trials. Table 2 provides a comprehensive overview of the evaluation procedure employed for the trials using the Cochrane risk-of-bias assessment tool.

**Table 2:** Quality appraisal using the Cochrane risk-of-bias assessment tool

Cochrane risk-of-bias assessment tool [8]	Sands et al. [9]	Rutgeerts et al. [10]	Kucharzik et al. [11]	Feagen et al. [12]	Vermeire et al. [13]
Random sequence generation	+	+	+	+	+
Allocation concealment	+	-	+	+	-
Blinding of participants and personnel	+	+	+	+	+
Blinding of outcome assessment	+	+	+	+	+
Incomplete outcome data	+	-	+	-	-
Selective reporting	+	+	+	+	+
Other bias	+	+	+	+	+

Table 3 provides a summary of the quality appraisal procedure employed for the case reports included in the study, using the Joanna Briggs Institute (JBI) critical appraisal checklist for case reports.

**Table 3:** Quality assessment of the case reports

Critical Appraisal Checklist for Case Reports [14]	Smeets et al. [15]				Costa. et al. [16]			
Were the patient's demographic characteristics clearly described?	Yes				Yes			
Was the patient's history clearly described and presented as a timeline?	Yes				Yes			
Was the current clinical condition of the patient on presentation clearly described?	Yes				Yes			
Were diagnostic tests or assessment methods and the results clearly described?	Yes				Yes			
Was the intervention(s) or treatment procedure(s) clearly described?	Yes				Yes			
Was the post-intervention clinical condition clearly described?	Yes				Yes			
Were adverse events (harms) or unanticipated events identified and described?	No				No			
Does the case report provide takeaway lessons?	Yes				Yes			

The eligibility of observational studies was evaluated by employing appropriate quality rating tools. The findings of the quality appraisal are presented in Table 4.

**Table 4:** Quality appraisal using the Newcastle-Ottawa Scale

Study	Selection	Compatibility	Outcome
Yao et al. [17]	****	*	***
Lenti et al. [3]	****	**	***
Brewer et al. [18]	****	**	***

 The quality assessment procedure for the systematic reviews is outlined in Table 5.

**Table 5:** Quality appraisal using AMSTAR checklist AMSTAR - Assessment of Multiple Systematic Review

AMSTAR checklist [19]	Engel et al. [20]	Meserve et al. [21]
Did the research question and inclusion criteria for the review include components of PICO?	yes	Yes
Did the review report contain an explicit statement that the review methods were established prior to the review, and did the report justify any significant deviations from the protocol?	Yes	Yes
Did the review authors explain their selection of the study designs for inclusion in the review?	Yes	Yes
Did the review authors use a comprehensive literature search strategy?	Yes	Yes
Did the review authors perform study selection in duplicate?	No	No
Did the review authors perform data extraction in duplicate?	No	No
Did the review authors provide a list of excluded studies and justify the exclusions?	Yes	Yes
Did the review authors describe the included studies in adequate detail?	Yes	Yes
Did the review authors use a satisfactory technique for assessing the risk of bias (RoB) in individual studies that were included in the review?	No	Yes
Did the review authors report on the sources of funding for the studies included in the review?	No	No
If meta-analysis was performed, did the review authors use appropriate methods for the statistical combination of results?	Yes	Yes
If meta-analysis was performed, did the review authors assess the potential impact of RoB in individual studies on the results of the meta-analysis or other evidence synthesis?	Yes	No
Did the review authors account for RoB in individual studies when interpreting/ discussing the results of the review?	No	Yes
Did the review authors provide a satisfactory explanation for and discussion of any heterogeneity observed in the results of the review?	Yes	Yes
If they performed quantitative synthesis, did the review authors carry out an adequate investigation of publication bias (small study bias) and discuss its likely impact on the results of the review?	Yes	Yes
Did the review authors report any potential sources of conflict of interest, including any funding they received for conducting the review?	Yes	No
Total score out of 16	12/16	11/16

Outcomes Measured

The primary outcome studied from the finalized research papers was to assess the safety and efficacy of ustekinumab and vedolizumab in treating inflammatory bowel disease. The secondary outcome extracted was the efficacy of both drugs as induction and maintenance treatment for IBD, also including their safety profiles.

Study Characteristics

A total of 12 research papers were reviewed, including 5681 participants. Out of the papers selected, there were five randomized controlled trials, two systematic reviews and meta-analyses, two case reports, and three observational studies. All studies involved patients with IBD, who were either biologic therapy-naive or lacked a response to the biologic therapies. One of the trials showed that ustekinumab proved to be an effective therapy for induction and maintenance treatment in UC patients compared to placebo; two other trials exhibited the effectiveness of ustekinumab in remission of transmural inflammation and endoscopic healing by showing a reduction in the simplified endoscopic score for Crohn's disease (SES-CD) in patients of treatment-refractory Crohn's disease. In contrast, other studies proved it effective in treating perianal CD. The case reports described the leukocytoclastic reaction and Large T-cell lymphoma in patients being treated with ustekinumab over the course of their treatment. Still, no causal link was found to be associated with it since several other factors could have led to these reactions. A post hoc analysis and randomized controlled trial (RCT) proved vedolizumab to be an effective treatment for IBD in biologic naive patients compared to a placebo. At the same time, a systematic review also signified the efficacy of vedolizumab as an IBD treatment in induction and maintenance. Table 6 summarizes the findings of the studies included in the review.

**Table 6:** Summary of included studies CD - Crohn's disease; IBD - inflammatory bowel disease; UC - ulcerative colitis; SES-CD - simplified endoscopic activity score for Crohn's disease; IUS - intestinal ultrasound; BWT - bowel wall thickness; TNF - tumor necrosis factor; SC - subcutaneously; ALK - anaplastic lymphoma kinase; LV - leukocytoclastic vasculitis; UST - ultrasound therapy; RCT - randomized controlled trial

Author and year	Type of study	Purpose of study	Number of participants	Results	Conclusion
Lenti et al., 2018 [3]	Observational study (cohort)	To study vedolizumab-treated IBD patients' clinical outcomes and safety	203	203 IBD patients were observed for 16±8 months. Among them, 135 had CD, and 68 had UC. At 14 weeks, CD patients showed a clinical response/remission of 78.5%, while UC patients had a response/remission rate of 91.2%. At 52 weeks, CD and UC patients had response/remission rates of 63.9% and 82.5%, respectively. The adherence rate was high at 97%. The cumulative incidence of infectious illnesses was 11.9 per 100 person-years.	The study concluded that vedolizumab is an effective therapy for inducing and maintaining remission of IBD.
Sands et al., 2019 [9]	Randomized controlled trial	To study the efficacy of ustekinumab as induction and maintenance therapy in patients with ulcerative colitis	961	Patients who received IV ustekinumab in the induction phase (8 weeks) and maintenance (44 weeks) had higher clinical remission rates of UC (15.5% and 43.8%) compared to those who were given a placebo (5.3% and 24%, respectively) with no difference in adverse effects observed between both groups.	In this trial involving patients with moderate to severe UC, despite previous treatment with or without biologics, ustekinumab was more effective in inducing and maintaining remission than placebo.
Rutgeerts et al., 2018 [10]	Randomized controlled trial	To study the effectiveness of ustekinumab in promoting endoscopic healing among individuals diagnosed with Crohn's disease	334	Patients receiving ustekinumab had a greater reduction in simplified endoscopic activity score for Crohn's disease (SES-CD) than those receiving a placebo at both the induction and maintenance phases of the trial.	The findings from three clinical trials involving patients diagnosed with moderate to severe Crohn's disease indicate that the administration of ustekinumab by intravenous induction and subcutaneous maintenance leads to a significant reduction in SES-CD compared to using a placebo. Significant reductions were seen in endoscopic disease activity at week 8 of induction therapy.
Kucharzik et al., 2023 [11]	Randomized Controlled Trial	To investigate the early ultrasonography response and progressive transmural remission following therapy with ustekinumab in individuals diagnosed with Crohn's disease	77	Seventy-seven patients were studied in this trial by intestinal ultrasound (IUS) starting at four weeks and followed till 48 weeks of Ustekinumab therapy. At 48 weeks, IUS response and transmural remission were 46.3% and 24.1%, respectively; IUS response, BWT, and transmural remission were more pronounced in biologic naive patients.	This multicentre, international, and interventional trial studying the effects of ustekinumab-treated CD patients showed IUS response and transmural remission at 48 weeks, with a more significant response to biologics in naive patients.
Feagen et al., 2019 [12]	Post hoc analysis of randomized controlled trial	To study the response of vedolizumab in biologic therapy-naive patients of IBD	1158 (374 CD patients and 784 UC patients)	At the end of the second week, 19.1% of vedolizumab-treated ulcerative colitis (UC) patients had favorable rectal bleeding and stool frequency scores. The placebo group had 10% overall and 6.6% TNF antagonist naïve. In TNF antagonist-naive patients with Crohn's disease (CD), vedolizumab showed higher response rates for abdominal pain and stool frequency at both week 2 (15.0% vs. 7.9% on placebo) and week 4 (23.8% vs. 10.3% on placebo).	In a post-hoc analysis of phase 3 trials, vedolizumab showed early and significant improvement in patient-reported symptoms of UC and CD as early as week 2; the improvement continued to week 6. This effect was particularly notable when vedolizumab was used as the first-line biologic therapy.
Vermeire et al., 2021 [13]	Randomized controlled trial	To study the efficacy of subcutaneous vedolizumab in the treatment of CD	410	Following vedolizumab intravenous induction, patients were randomized to receive vedolizumab subcutaneously (SC) or placebo maintenance. At Week 52, vedolizumab SC showed higher rates of clinical remission (48.0% vs. 34.3%, p=0.008) and corticosteroid-free clinical remission (45.3% vs. 18.2%) compared to placebo. Injection site reactions were the only notable safety concern (2.9%).	This trial supports subcutaneous vedolizumab as an effective treatment for Crohn's disease. Higher clinical remission rates, including corticosteroid-free remission, indicate its substantial clinical benefits. While injection site reactions were observed, vedolizumab SC maintains an overall favorable safety profile, reinforcing its value in treating Crohn's disease.
Smeets et al., 2019 [15]	Case report	Case of large T cell lymphoma in a patient with therapy refractive CD taking ustekinumab	1	29-year-old female with refractory CD was treated with multiple trials of immunotherapy. She developed paraaortic, para iliac, and retroperitoneal lymphadenopathy, which on biopsy, demonstrated to be an anaplastic large cell ALK-positive lymphoma treated with multiple chemotherapy cycles.	After analysis, similar case reports and literature showed that some malignancy cases were reported after using ustekinumab. Still, the statistical incidence of these was no different than the normal population, and there were multiple risk factors in this case, like multiple biologic therapies that could have led to the malignancy. Hence, no causal link was found to ustekinumab therapy.
Costa-Moreira et al., 2020 [16]	Case report	Case of leukocytoclastic vasculitis in a patient treated with ustekinumab	1	A 23-year-old patient being treated with ustekinumab for three years presented with LV, was treated with steroids, and restarted ustekinumab, leading to a new episode of LV.	An analysis of literature and similar case reports showed association of LV with biologic therapies can have latency periods of several years, coinciding with findings in this case, i.e., developing lV after three years of ustekinumab therapy.
Yao et al., 2021 [17]	Retrospective cohort study	To study the effects of ustekinumab through concentration on clinical and endoscopic outcomes in patients of refractory CD	19	A total of nineteen individuals who met the eligibility criteria were included in the study. The average age of these patients was 29.1±9.1 years, and the average duration of their disease was 5.5±4.7 years. At the 16th week out of a total of 20 weeks following the initiation of UST treatment, the rates of clinical response, clinical remission, endoscopic response, and endoscopic remission were found to be 89.5%, 84.2%, 42.2%, and 73.7% respectively. A UST trough concentration >1.12 μg/mL was associated with a significantly higher rate of endoscopic remission (70.0% vs. 11.1%, p=0.02)	Ustekinumab proved to be an effective treatment for refractory CD, UST trough concentration of >1.12 ug/ml was associated with endoscopic remission of the disease at 16/20 weeks.
Brewer et al., 2021 [18]	Retrospective cohort study	To study the efficacy of ustekinumab in treating luminal and perianal Crohn's disease	27	At six months, 48.1% of patients (13/27) had a positive response in fistula condition, but no remission was achieved. Patient-reported symptomatic improvement was seen in 59.3% (16/27), with only 3.7% (1/27) achieving complete symptomatic remission. At one year, 55.6% (5/9) showed a response in fistula condition but no remission, while all nine patients (100%) experienced symptomatic improvement and 22.2% (2/9) achieved complete symptomatic remission.	Ustekinumab proved to be a promising treatment in the symptomatic treatment of perianal Crohn's disease, with 55.6% of patients achieving sustained response at 12 months follow-up and a good safety profile. However, further prospective studies are required to prove its efficacy.
Engel et al., 2017 [20]	A systematic review and pooled analysis	To study the safety and efficacy of vedolizumab in treatment induction and maintenance of IBD	1565	Nine studies comprising 1565 adult patients (571 with UC and 994 with CD) were analyzed. For CD, clinical response and remission rates at week 6 were 54% and 22%, respectively, increasing to 49% and 32% at week 14 and 45% and 32% at week 52. In UC, clinical response and remission rates at week 6 were 43% and 25%, respectively, rising to 51% and 30% at Week 14/22 and 48% and 39% at week 52. Adverse effects were mostly minor, observed in 30.6% of patients, while infections were reported in 3.4% of patients	The analysis of previous literature, RCT proved vedolizumab to be an effective induction and maintenance treatment for UC and CD.
Meserve et al., 2022 [21]	Systemic review and meta-analysis	To study the effectiveness of re-induction and dose escalation of ustekinumab in Crohn's disease patients who either lost response or had an inadequate response to ustekinumab	925	In the meta-analysis, ustekinumab dose escalation resulted in a clinical response for 55% of patients with inadequate or lost response. The endoscopic response was achieved by around 61% of patients, with 29% reaching endoscopic remission. Dose interval shortening alone restored response in 57% of patients. No consistent factors associated with response to dose escalation were found.	In a meta-analysis of cohort studies, ustekinumab re-induction and/or dose interval shortening effectively captured response in patients with CD with inadequate response, or loss of response, to initial induction and/or maintenance therapy.

Discussion

Inflammatory bowel disease is a group disorder that causes inflammation in the gastrointestinal tract. IBD includes two diseases, i.e., Crohn's disease and ulcerative colitis. There is no known etiology of this disease. Still, genetics, autoimmunity, and certain environmental triggers (e.g., smoking, stress, depression) are associated with a new incidence of IBD in patients with positive family history. Crohn's disease involves the whole gastrointestinal system, i.e., from mouth to anus, and causes transmural inflammation, whereas ulcerative colitis is mostly limited to the rectum involving the mucosa and submucosa.

Over the years, many therapies have been implemented to control and cure IBD, but no definite cure has been found; certain drugs have proved to be efficacious in controlling the symptoms and flare-ups of the disease. Commonly used therapies include amino salicylates, steroids, and immunomodulators like azathioprine and methotrexate.

Over the past decade, new drugs, i.e., biologic therapies like anti-TNF therapies, have been studied and introduced as a treatment for IBD for patients not responding to the drugs used or because of harmful side effects associated with the traditional therapies, have proved to be beneficial but refractory disease and a substantial number of non-responders still pose as a significant challenge.

Some of the new biologic therapies, like ustekinumab (an IL -23 inhibitor) and vedolizumab (a humanized monoclonal antibody), have been introduced as novel treatments for patients resistant to previous biologic therapies and refractory IBD.

In this systematic review, we have analyzed the literature and clinical trials to find out the safety profile and efficacy of ustekinumab and vedolizumab in the treatment of IBD and their efficacy in induction and maintenance of remission over the years as a secondary outcome of this review. Following is the summary of the findings discovered by carefully reviewing the selected papers.

Ustekinumab in Ulcerative Colitis (UC)

In a randomized controlled trial by Sands et al., patients receiving ustekinumab induction and maintenance therapy were randomly allocated to three groups: placebo (319 patients), ustekinumab 130 mg dose (320 patients), or ustekinumab at approximately 6 mg per kilogram (322 patients). Patient characteristics were comparable among the different trial groups in the induction and maintenance phases. The results showed significantly higher clinical remission rates (15.5% and 43.8%) compared to the placebo group (5.3% and 24%) [9]. No significant difference in adverse effects was observed between the ustekinumab and placebo groups.

Ustekinumab in Crohn's Disease (CD)

In a randomized controlled trial by Rutgeerts et al., the administration of ustekinumab for both induction and maintenance therapy demonstrated a more substantial decrease in the Simplified Endoscopic Disease Activity Score for Crohn's disease (SES-CD) compared to the placebo group [10]. Specifically, patients receiving ustekinumab experienced a reduction of 2.8 points from the baseline at induction until week 8, whereas patients in the placebo group only experienced a reduction of 0.7 points [10]. In a retrospective cohort study by Brewer et al., ustekinumab showed promise in treating symptomatic perianal Crohn's disease, with 55.6% of patients achieving sustained response at 12 months in patients with refractory Crohn's disease [18].

Vedolizumab in Inflammatory Bowel Disease (IBD)

A post-hoc analysis by Feagen et al. (2019) revealed that vedolizumab demonstrated early and significant improvement in patient-reported symptoms of UC and CD, with notable response rates observed as early as week 2 [12]. Similarly, in a randomized controlled trial by Vermeire et al. (2021), patients receiving vedolizumab subcutaneously showed higher rates of clinical remission and corticosteroid-free clinical remission at Week 52 compared to the placebo group [13].

Safety and Efficacy

Overall, both ustekinumab and vedolizumab demonstrated efficacy in inducing and maintaining remission in patients with UC and CD, with higher percentages of clinical remission and endoscopic mucosal healing [10]. Treatment with ustekinumab partially improved Mayo scores, and CRP, lactoferrin, and calprotectin recorded at baseline in the maintenance trial in the group treated with ustekinumab. In contrast, results for these measures worsened in the placebo group [9]. Adverse effects were mostly minor and comparable between treatment groups in most studies. Both the case reports presenting a leukocytoclastic reaction and Large T cell lymphoma in patients being treated with ustekinumab failed to provide any promising evidence of ustekinumab being the causative factor, as many other confounding factors would have led to these conditions in addition to ustekinumab. [16,15]. No consistent factors associated with response to Ustekinumab dose escalation were found in the meta-analysis by Meserve and co-authors [21]. Injection site reactions were the only notable safety concern associated with vedolizumab in the study by Vermeire et al. [13].

Differences Between Ustekinumab and Vedolizumab

Both ustekinumab and vedolizumab demonstrated efficacy in specific subsets of patients, with differences in response rates and remission rates observed in CD and UC. Ustekinumab showed higher clinical remission rates in UC compared to placebo. In contrast, vedolizumab demonstrated a better response in patients with TNF antagonist-naive CD in the study by Feagen et al. [12]. 

Comparison With Conventional Therapies

Ustekinumab and vedolizumab are emerging as safe and effective therapies for treating IBD; previously, anti-TNF agents also proved to be efficacious in decreasing surgery and hospitalization rates in both UC and CD patients [22]. Nevertheless, a significant proportion of patients, around one-third, exhibit no response to anti-tumor necrosis factor (anti-TNF) treatments. Approximately 40% of patients who initially exhibit a positive response eventually cease to respond due to factors such as subtherapeutic drug levels, the emergence of antidrug antibodies, or the occurrence of mechanistic escape, wherein another cytokine may assume more significance in the pathogenesis of the disease [23]. In addition, it is worth noting that a minority of patients may necessitate a modification in their treatment regimen as a result of experiencing side effects, such as drug-induced lupus, psoriasis, or demyelinating disease.

Compared to standard anti-TNF medicines, vedolizumab demonstrates a relatively low level of immunogenicity. When comparing patients who were administered concomitant immunosuppressant at baseline with those who were not, there was a slight decrease in the chance of producing anti-vedolizumab antibodies, with rates of 3% and 4%, respectively [24].

The immunogenicity associated with ustekinumab is notably minimal. The CERTIFI study observed that 0.7% of participants developed antibodies to ustekinumab by week 36. The maintenance IM-UNITI trial revealed a higher percentage of 2.3% by the end of the first year [25]. The observed rates are significantly lower in comparison to anti‐TNF medicines, such as infliximab (ranging from 0% to 83%), adalimumab (ranging from 0% to 54%), and golimumab (ranging from 0% to 19%) [26]. There is significant anticipation for the release of long-term immunogenicity data pertaining to ustekinumab.

Factors Influencing Treatment Outcomes

The trial's patient selection criteria primarily focused on key factors such as biologic therapy status, disease duration, and severity. Specifically, patients considered were either biologic therapy-naive or non-responders to previous biologics, and some had a history of resistance to all prior treatments. The impact of simultaneous treatment with corticosteroids and immunomodulators on vedolizumab therapy in IBD patients was also investigated [3]. These comprehensive variables aimed to ensure a diverse and relevant patient population, providing valuable insights into the safety and efficacy of the treatments under study. As observed in the study by Feagen and co-authors, vedolizumab proved more beneficial in TNF antagonist naive patients [12].

Recommendations

The findings suggest that ustekinumab and vedolizumab are effective treatment options for IBD patients with inadequate response or loss of response to previous therapies. Further research is needed to identify factors influencing response to dose escalation in ustekinumab-treated CD patients. Both biologics showed a good safety profile, but long-term follow-up and larger studies are required to better understand their safety profiles in real-world settings.

Limitations

The identified studies contained trials that were not long enough to fully capture the efficacy of ustekinumab in the treatment of UC over the years, and the studies did not include head-to-toe comparisons with other biologic therapies. Trials that include the pediatric patient population were not included in the papers reviewed. Despite these limitations, the studies collectively provide valuable insights into the safety and efficacy of ustekinumab and vedolizumab in treating IBD.

## Conclusions

The reviewed studies provide strong evidence for the safety and efficacy of ustekinumab and vedolizumab in treating IBD. However, individual patient characteristics, disease subtypes, and previous treatments may influence therapeutic response, highlighting the need for personalized treatment approaches in IBD management. Altogether, the clinical trials prove the effectiveness of ustekinumab and vedolizumab compared to placebo by significant results. Overall, ustekinumab and vedolizumab hold great promise as valuable therapeutic options for patients with IBD, but further research is warranted to optimize treatment strategies and improve.
