# Correction: Safety and Efficacy of Biologic Therapies (Ustekinumab and Vedolizumab) in the Treatment of Inflammatory Bowel Disease (IBD): A Systematic Review

**DOI:** 10.7759/cureus.c318

**Published:** 2025-09-24

**Authors:** Hafsa Ashraf, Adiprasad Bodapati, Ayesha Hanif, Donatus K Okafor, Gitika Katyal, Gursharan Kaur, Safeera Khan

**Affiliations:** 1 Internal Medicine, California Institute of Behavioral Neurosciences & Psychology, Fairfield, USA; 2 Medicne, California Institute of Behavioral Neurosciences & Psychology, Fairfield, USA; 3 Research, California Institute of Behavioral Neurosciences & Psychology, Fairfield, USA

This article has been corrected to fix the following typos in the Study Identification and Selection paragraph: 29 articles (not 24) were shortlisted and put through quality assessment.

